# Supplementary material for: Disinhibition enables vocal repertoire expansion after a critical period
Source: Nat Commun. 2024 Aug 31;15:7565. doi: 10.1038/s41467-024-51818-4 (PMC11365960; doi:10.1038/s41467-024-51818-4)
Supplement: Supplementary file 1 — Supplementary Information [file 41467_2024_51818_MOESM1_ESM.pdf]

Supplementary Information File:

Disinhibition enables vocal repertoire expansion after a critical period

Fabian Heim[1], Ezequiel Mendoza[1,2], Avani Koparkar[1,3,4], Daniela Vallentin[1a]

[1] Max Planck Institute for Biological Intelligence, 82319 Seewiesen, Germany

[2] present address: Freie Universität Berlin, Takustr. 6, 14195 Berlin, Germany

[3] Indian Institute of Science Education and Research (IISER), Pune, India

[4] present address: Eberhard-Karls-Universität Tübingen, Germany

[a] To whom correspondence may be addressed:

Daniela Vallentin

Max Planck Institute for Biological Intelligence

Eberhard-Gwinner-Str. 11

82319 Seewiesen, Germany

daniela.vallentin@bi.mpg.de

Supplementary Figure 1

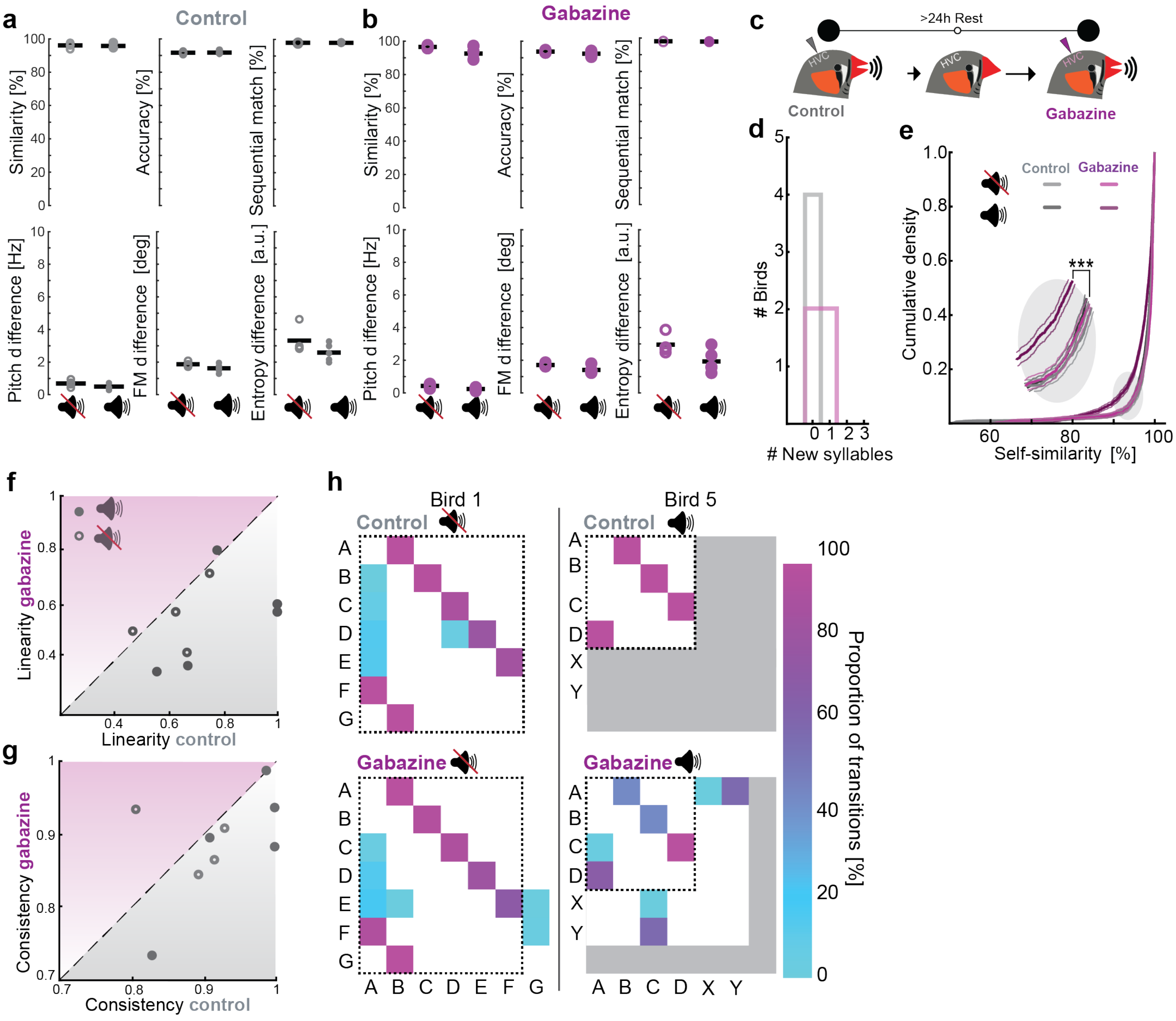

Supplementary Figure 1 | a) Song spectral features during control infusions for the playback and no- playback conditions. **b)** Song spectral features during gabazine application with and without playbacks. **c)** Experimental timeline: Adult zebra finch song was recorded while PBS was bilaterally infused in HVC. After a 24-hour rest period the same experiment was repeated while gabazine was applied. **d)** Number of new syllables produced in the control condition (when PBS was infused (grey)) and during gabazine infusion (purple) without playback. **e)** Empirical cumulative density curves of self-similarity comparisons for control and gabazine conditions when different groups of birds were either exposed to playback (+playback) or in the absence of playback (-playback). Thin lines indicate the 95% confidence interval for each condition. **f)** Song linearity during the control or gabazine condition for n = 5 birds in the presence (filled circles) or n=4 birds in the absence (open circles) of playback. **g)** Song

consistency during the control or gabazine condition in the presence or absence of playback as in f). **h)** Matrix plots illustrating syllable transition proportions for two birds under control and gabazine conditions in the presence or absence of playback. Dashed line indicates the pre-existing syllables.

Supplementary Figure 2

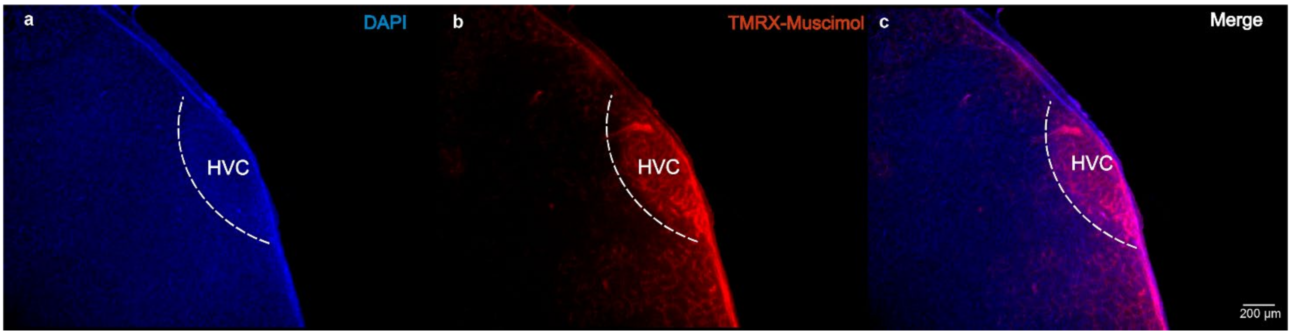

Supplementary Figure 2 | Spread of a pharmacological agent. a) DAPI staining of HVC. **b)** Fluorescent muscimol highlighted red. **c)** Merge between **a)** and **b)**.

Supplementary Figure 3

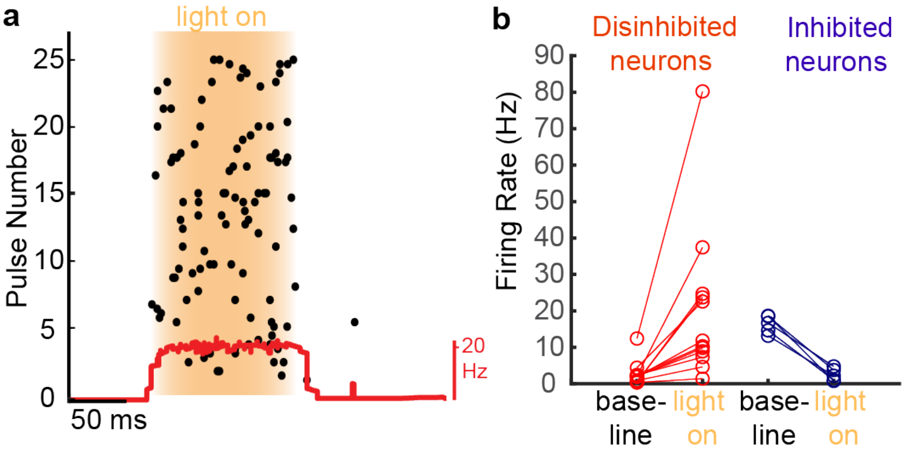

Supplementary Figure 3 | Effect of light stimulation on HVC neurons in ArchT+ birds. a) Spike raster plot of an example unit in HVC of an ArchT+ bird, disinhibited during pulses of optogenetic stimulation (yellow shaded area). **b)** Firing rates during baseline and light stimulation of significantly disinhibited and inhibited HVC neurons in ArchT+ birds. In addition to the 5/78 neurons that significantly decreased their firing rate during light stimulation, we also recorded 15/78 neurons that significantly increased their firing rate suggesting a direct disinhibitory effect on these neurons. Overall firing rates during baseline of disinhibited HVC neurons (median 2.034 Hz) was lower than of inhibited HVC neurons (median 16.698 Hz, p= 3.2321e-04, Wilcoxon rank sum test). Since HVC projection neurons exhibit a lower resting spiking activity compared to interneurons [47] this results supports the notion that the disinhibited neurons were mainly putative HVC projection neurons.

Supplementary Figure 4

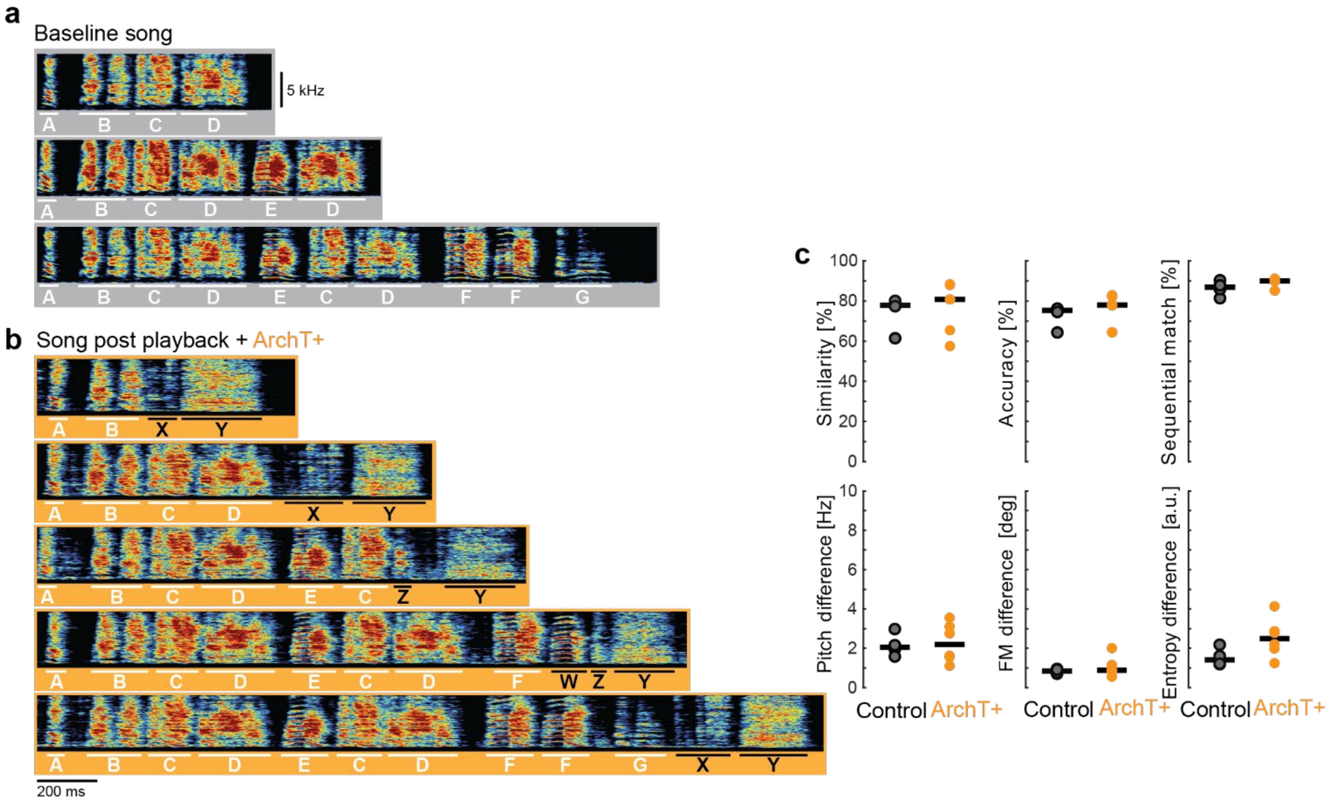

Supplementary Figure 4 | Example song recordings of one bird during baseline and at the final stage when exposed to αβ-playback. a) Multiple motifs of variable duration produced by ArchT+ bird 3 during baseline recordings prior to playback and optogenetic stimulation. **b)** Multiple motifs of variable duration produced by the same bird as in a) four weeks after playbacks had ended. **c)** Song similarity, accuracy and sequential match of baseline songs versus songs (core motif) produced at the final stage after optogenetic and playback exposure. Pitch difference, frequency modulation difference and entropy difference of the songs produced during baseline and final song recordings.

Supplementary Figure 5

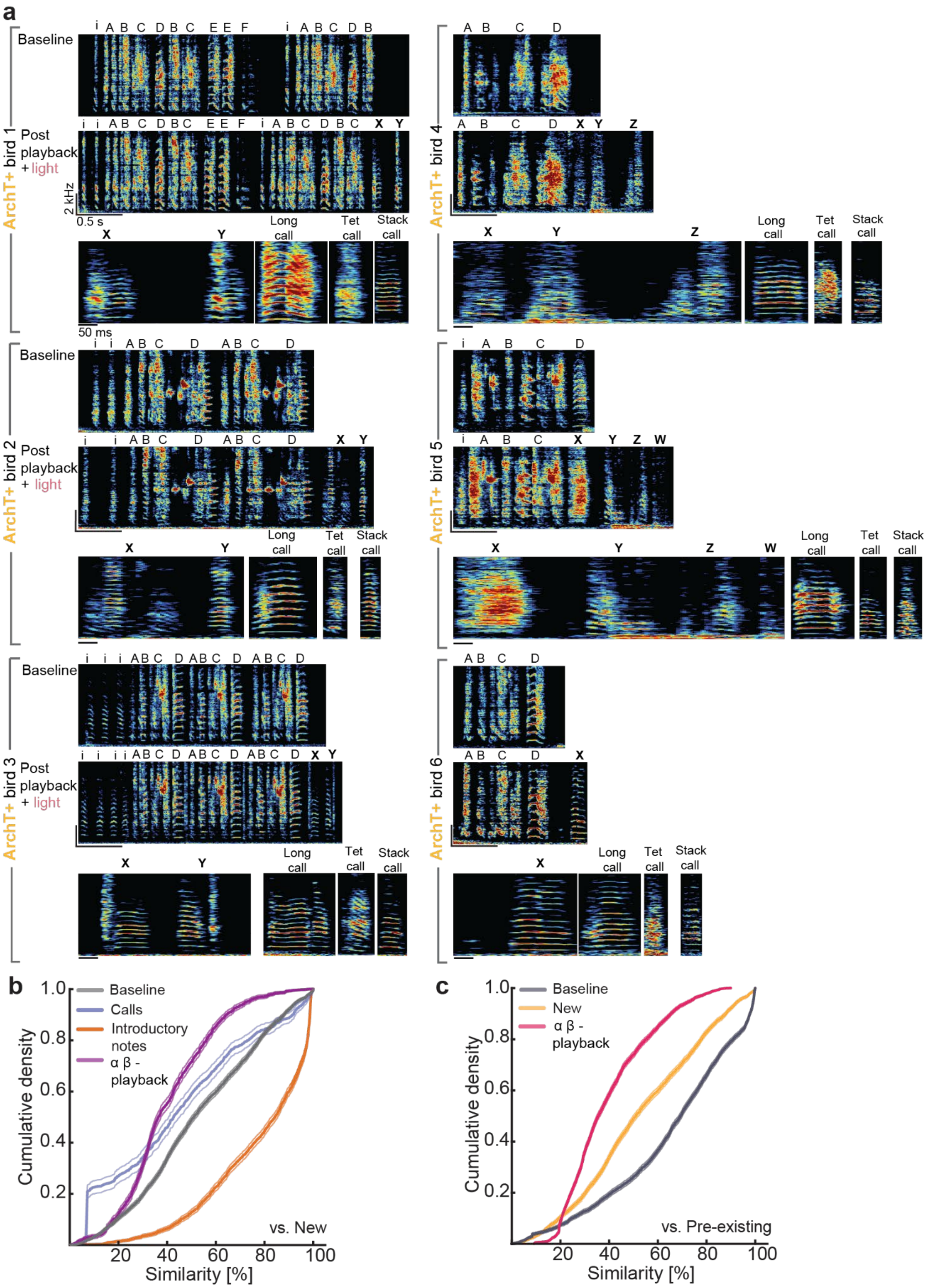

Supplementary Figure 5 | Comparisons of songs of optogenetically manipulated birds produced at baseline and after stimulation versus the individual vocal repertoire. a) Top panel for each bird shows a representative motif at baseline, middle panel a motif during the final phase. Bottom panel displays the exemplary novel syllables as well as representative long, tet and stack calls of each bird. **b)** Empirical cumulative density of cross-similarity comparisons between baseline syllables (grey) calls (blue), introductory notes (yellow) and playback syllables (purple) versus new syllables. **c)** Empirical cumulative density curves of cross-similarity comparisons between all pre-existing syllables within the same bird (grey), pre-existing versus new syllables within the same bird (yellow), and playback syllables versus new syllables (pink). $\chi^2 = 445.1119$, $p = 3.7683\times10^{-9}$, Kruskal-Wallis test.

Supplementary Figure 6

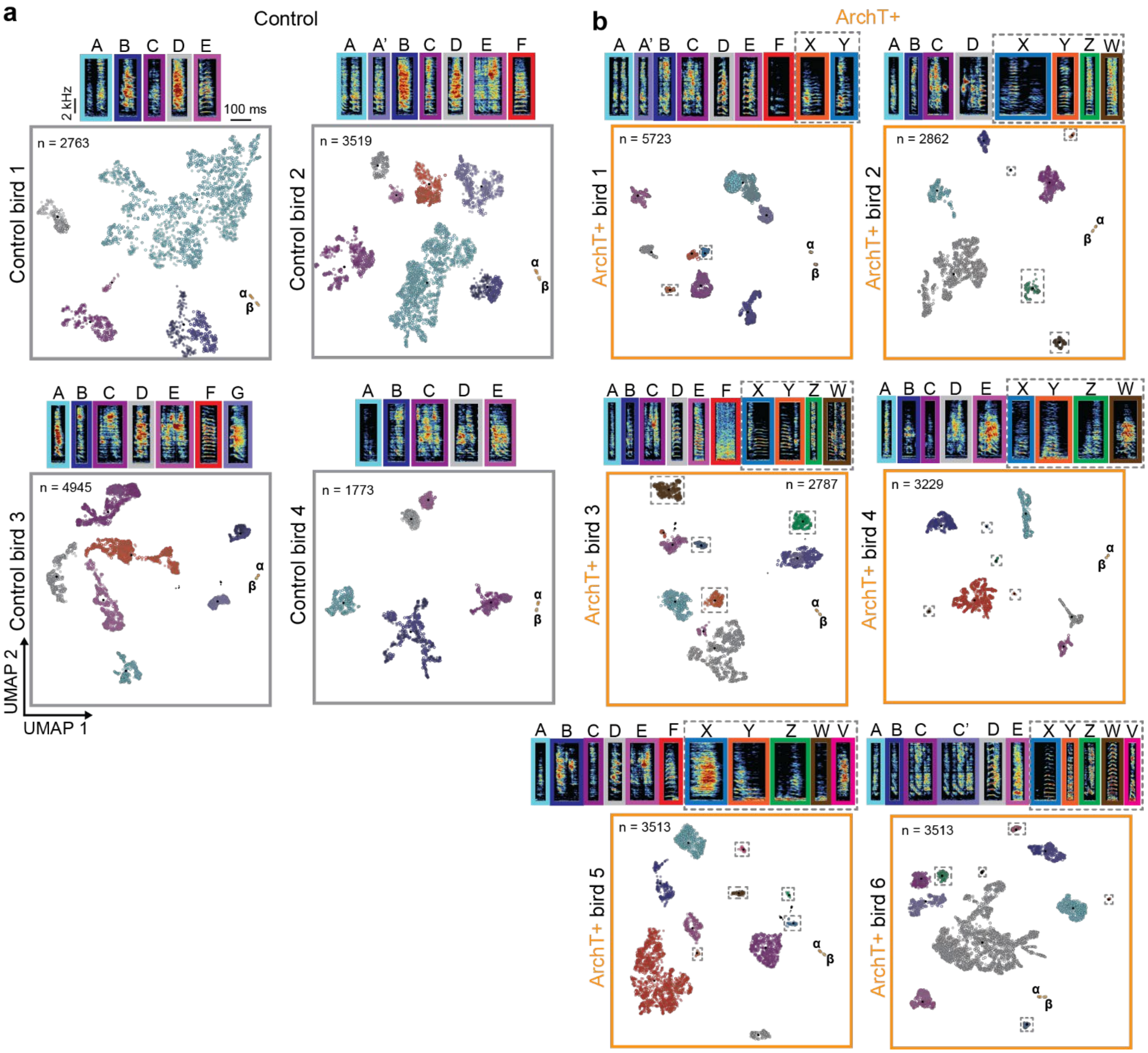

Supplementary Figure 6 | UMAP dimensionality reduction with HDBScan clustering of segmented syllables of all birds. a) HDBScan clustering for control birds. Different coloured circles correspond to sonogram of representative syllables highlighted with the same colour on the top. Open circles are the syllables produced at baseline whereas filled circles correspond to syllables produced at the final stage. n indicates the number of syllables that were segmented with DAS for each individual bird. α, β indicate playback syllables. **b)** HDBscan clustering for ArchT+ birds. New syllables that represent a new cluster at the final stage are highlighted with a dotted grey line.

Supplementary Figure 7

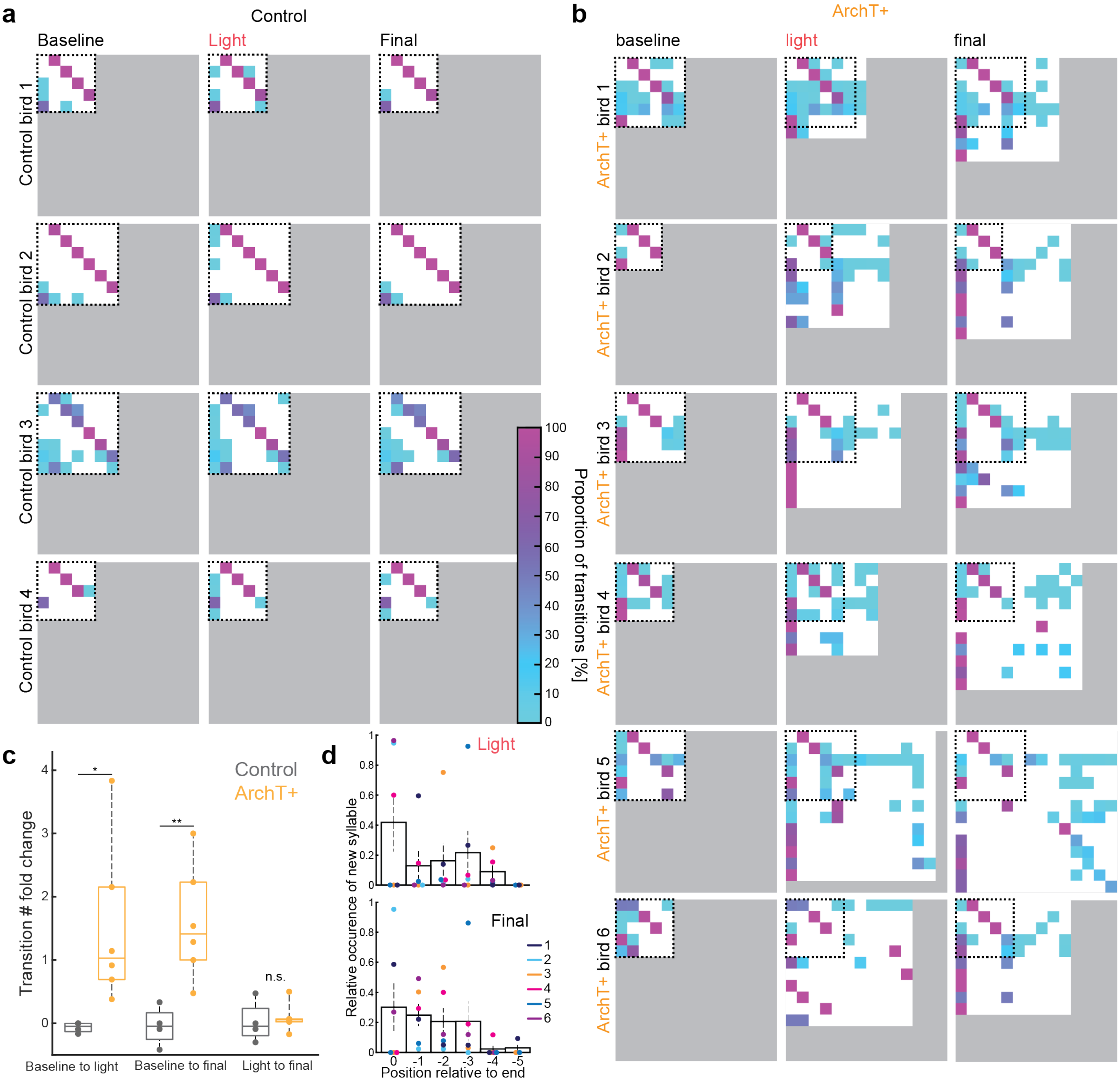

Supplementary Figure 7 | Optogenetically manipulated birds add novel syllables at the end of their motif. a) Matrix plots illustrating syllable transitions from low (turquoise) to high (purple) proportions. Matrices are constructed with data from three different timepoints throughout the experiment for all control (a) and ArchT+ injected birds (**b**). Baseline visualises transitions prior to the playback and stimulation epoch, light condition shows transition data based from recordings at the end of the four-week playback and stimulation epoch, 'final' represents transitions four weeks after the stimulation epoch. **c)** Population level data highlights increased number of syllable transitions between recording epochs in ArchT+ but not control birds. The number of transitions is significantly increased between ArchT+ birds and control birds between pre-playback (baseline) after the playbacks had ended ($p = 0.0139$, Wilcoxon rank sum). The relative number of transitions is increased in ArchT+ birds between pre-playback and post-playback recordings ($p = 0.0095$, Wilcoxon rank sum). The number of transitions stays stable during the period between the end of the playback phase and four weeks after the playbacks had ended ($p = 0.3524$, Wilcoxon rank sum). **d)** Relative positioning of the occurrence of new syllables in relation to the end of ArchT+ birds' motifs separated by songs sung right after the playbacks had ended (light, upper panel) and four weeks after playbacks had ended (final, lower panel). Bird ID is colour coded and each dot represents the relative occurrence of a new syllable at the respective position. Error bars indicate standard error of the mean. Bars represent mean relative occurrence across all six ArchT+ birds.

Supplementary Figure 8

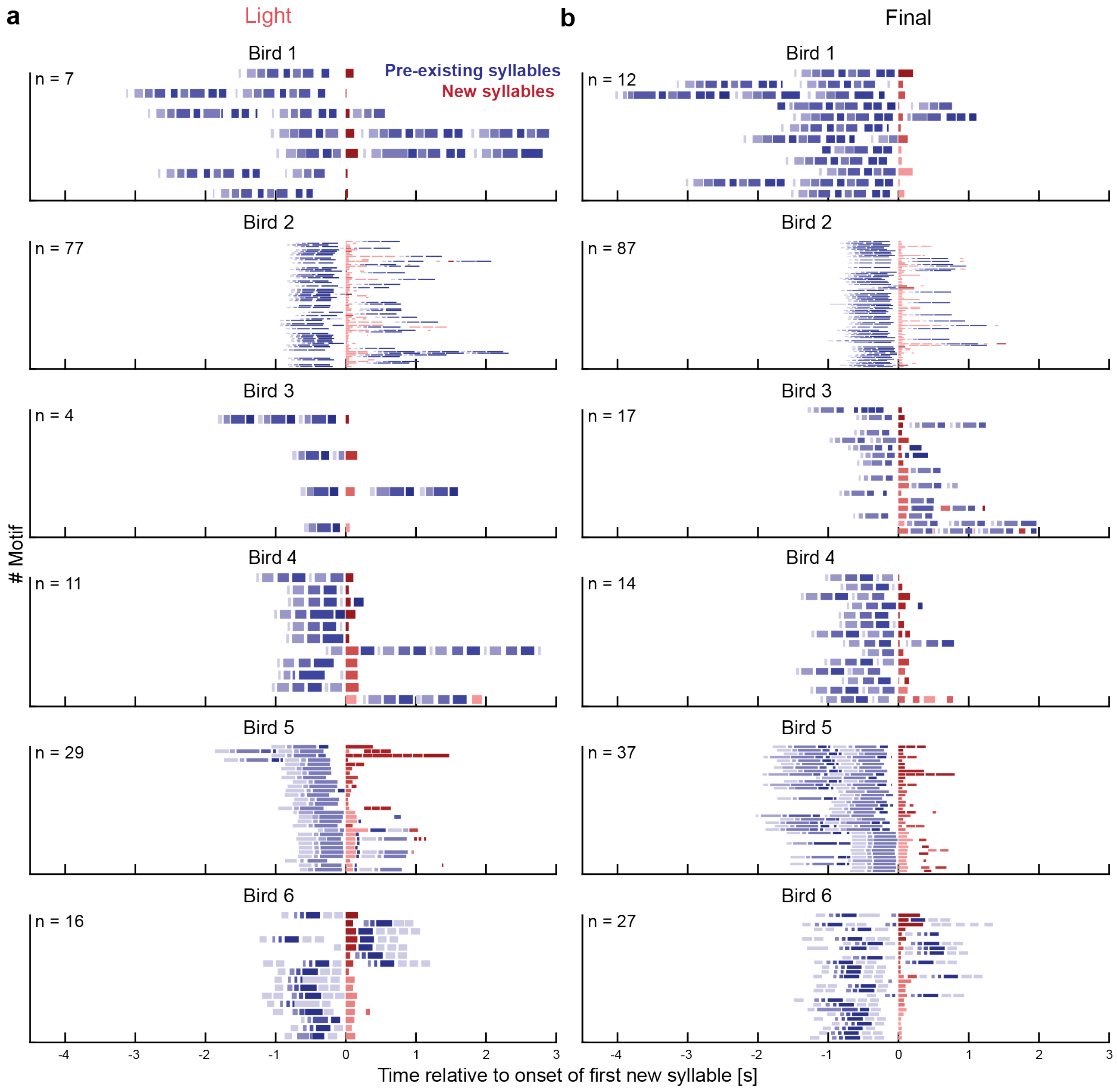

Supplementary Figure 8 | ArchT+ birds produce novel syllables towards the end of their motifs. a) Line plots illustrating the occurrence of novel syllables for each ArchT+ bird within the first 50 song bouts directly a) at the end of the light stimulation epoch (light) or **b)** four weeks after the playbacks had ended (final). Each line corresponds to one motif containing pre-existing syllables (blue shades) and novel elements (red shades). Individual syllables are shown as coloured lines and aligned to the onset of the first novel element.
