# Supplementary material for: Disinhibition enables vocal repertoire expansion after a critical period
Source: Nat Commun. 2024 Aug 31;15:7565. doi: 10.1038/s41467-024-51818-4 (PMC11365960; doi:10.1038/s41467-024-51818-4)
Supplement: Supplementary file 3 — Reporting Summary [file 41467_2024_51818_MOESM3_ESM.pdf]

nature portfolio

Corresponding author(s): Daniela Vallentin

Last updated by author(s): Jul 24, 2024

Reporting Summary

Nature Portfolio wishes to improve the reproducibility of the work that we publish. This form provides structure for consistency and transparency in reporting. For further information on Nature Portfolio policies, see our Editorial Policies and the Editorial Policy Checklist.

Please do not complete any field with "not applicable" or n/a. Refer to the help text for what text to use if an item is not relevant to your study.
For final submission: please carefully check your responses for accuracy; you will not be able to make changes later.

Statistics

For all statistical analyses, confirm that the following items are present in the figure legend, table legend, main text, or Methods section.

n/a	Confirmed	
☐	☒	The exact sample size (n) for each experimental group/condition, given as a discrete number and unit of measurement
☐	☒	A statement on whether measurements were taken from distinct samples or whether the same sample was measured repeatedly
☐	☒	The statistical test(s) used AND whether they are one- or two-sided<br>*Only common tests should be described solely by name; describe more complex techniques in the Methods section.*
☒	☐	A description of all covariates tested
☐	☒	A description of any assumptions or corrections, such as tests of normality and adjustment for multiple comparisons
☐	☒	A full description of the statistical parameters including central tendency (e.g. means) or other basic estimates (e.g. regression coefficient) AND variation (e.g. standard deviation) or associated estimates of uncertainty (e.g. confidence intervals)
☐	☒	For null hypothesis testing, the test statistic (e.g. F, t, r) with confidence intervals, effect sizes, degrees of freedom and P value noted<br>*Give P values as exact values whenever suitable.*
☒	☐	For Bayesian analysis, information on the choice of priors and Markov chain Monte Carlo settings
☒	☐	For hierarchical and complex designs, identification of the appropriate level for tests and full reporting of outcomes
☐	☒	Estimates of effect sizes (e.g. Cohen's d, Pearson's r), indicating how they were calculated

Our web collection on statistics for biologists contains articles on many of the points above.

Software and code

Policy information about availability of computer code

Data collection	Behavioral data was collected using Matlab 2013b 32-bit version and Sound Analysis Pro (SAP) 2011 Version 2011.104, Extracellular recordings were collected using Matlab 2013b 32-bit version. All custom made scripts will be made available on our Github once granted for publication.
Data analysis	Deep Audio Segmenter (DAS), Sound Analysis Pro (SAP) 2011 Version 2011.104 and Matlab 2018b was used to analyze all behavioral and extracellular recording data. All custom made scripts will be made available on our Github once granted for publication.

For manuscripts utilizing custom algorithms or software that are central to the research but not yet described in published literature, software must be made available to editors and reviewers. We strongly encourage code deposition in a community repository (e.g. GitHub). See the Nature Portfolio guidelines for submitting code & software for further information.

Data

Policy information about availability of data

All manuscripts must include a data availability statement. This statement should provide the following information, where applicable:

- Accession codes, unique identifiers, or web links for publicly available datasets
- A description of any restrictions on data availability
- For clinical datasets or third party data, please ensure that the statement adheres to our policy

Data Availability

Source data are provided with this paper. Example data are available here https://github.com/vallentinlab/HVC_disinhibtion. Due to space limitation of the public repository the complete song and neural recordings will be made available upon request. Please contact the corresponding author.

Code Availability
All custom scripts including example data are available here https://github.com/vallentinlab/HVC_disinhibtion.

Research involving human participants, their data, or biological material

Policy information about studies with human participants or human data. See also policy information about sex, gender (identity/presentation), and sexual orientation and race, ethnicity and racism.

Reporting on sex and gender	*Use the terms sex (biological attribute) and gender (shaped by social and cultural circumstances) carefully in order to avoid confusing both terms. Indicate if findings apply to only one sex or gender; describe whether sex and gender were considered in study design; whether sex and/or gender was determined based on self-reporting or assigned and methods used. Provide in the source data disaggregated sex and gender data, where this information has been collected, and if consent has been obtained for sharing of individual-level data; provide overall numbers in this Reporting Summary. Please state if this information has not been collected. Report sex- and gender-based analyses where performed, justify reasons for lack of sex- and gender-based analysis.*
Reporting on race, ethnicity, or other socially relevant groupings	*Please specify the socially constructed or socially relevant categorization variable(s) used in your manuscript and explain why they were used. Please note that such variables should not be used as proxies for other socially constructed/relevant variables (for example, race or ethnicity should not be used as a proxy for socioeconomic status). Provide clear definitions of the relevant terms used, how they were provided (by the participants/respondents, the researchers, or third parties), and the method(s) used to classify people into the different categories (e.g. self-report, census or administrative data, social media data, etc.) Please provide details about how you controlled for confounding variables in your analyses.*
Population characteristics	*Describe the covariate-relevant population characteristics of the human research participants (e.g. age, genotypic information, past and current diagnosis and treatment categories). If you filled out the behavioural & social sciences study design questions and have nothing to add here, write "See above."*
Recruitment	*Describe how participants were recruited. Outline any potential self-selection bias or other biases that may be present and how these are likely to impact results.*
Ethics oversight	*Identify the organization(s) that approved the study protocol.*

Note that full information on the approval of the study protocol must also be provided in the manuscript.

Field-specific reporting

Please select the one below that is the best fit for your research. If you are not sure, read the appropriate sections before making your selection.

☒ Life sciences ☐ Behavioural & social sciences ☐ Ecological, evolutionary & environmental sciences

For a reference copy of the document with all sections, see nature.com/documents/nr-reporting-summary-flat.pdf

Life sciences study design

All studies must disclose on these points even when the disclosure is negative.

Sample size	For pharmacology experiment:<br>Control group A: PBS infused in HVC or Gabazine infused in HVC + playback stimulation (n = 5 birds)<br>Control group B: PBS infused in HVC or Gabazine infused in HVC - playback stimulation (n=4 birds)<br><br>For optogenetics experiment:<br>Control group: Control-virus in HVC + playback stimulation (n=4 birds)<br>ArchT+ group: ArchT+ virus infused in HVC + playback stimulation (n=6 birds)<br><br>For electrophysiological virus functionality confirmation<br>Control group (n=3 birds)<br>ArchT+ group (n= 2 birds)<br><br>Sample size calculations were conducted in G*Power 3.1.9.7 based on an α of 0.05 and effect sizes observed during previous publications on altered song production.
Data exclusions	No data were excluded.
Replication	Each individual experiment was repeated multiple times including different birds or in different neurons and the results could be reliably replicated.
Randomization	The birds were assigned to the four groups by chance without prior pre-selection.

Blinding	Syllable labeling for the pharmacology experiment was done by a person blinded to the experimental conditions. The person had access to the songs and access to the bird ID but no access on the experimental condition.

Reporting for specific materials, systems and methods

We require information from authors about some types of materials, experimental systems and methods used in many studies. Here, indicate whether each material, system or method listed is relevant to your study. If you are not sure if a list item applies to your research, read the appropriate section before selecting a response.

Materials & experimental systems

n/a	Involved in the study
☐	☒ Antibodies
☒	☐ Eukaryotic cell lines
☒	☐ Palaeontology and archaeology
☐	☒ Animals and other organisms
☒	☐ Clinical data
☒	☐ Dual use research of concern
☒	☐ Plants

Methods

n/a	Involved in the study
☒	☐ ChIP-seq
☒	☐ Flow cytometry
☒	☐ MRI-based neuroimaging

Antibodies

Antibodies used	- primary antibodies against Parvalbumin (PV, 1:1000, mouse, PV 246, Swant, Burgdorf) and GFP (1:1000, rabbit, ab290, Abcam, Cambridge),<br>- donkey anti-mouse (1:200, A1037, Invitrogen, Waltham, MS) and donkey anti rabbit (1:200, A1042, Invitrogen).<br>- primary antibodies against PV, Calretinin (CR, 1:1000, goat, CG1, Swant, Burgdorf), Calbindin (CB, 1:1000, rabbit, CB38, Swant, Burgdorf) and eYFP (1:400, chicken, A10262, Thermo Fisher Scientific, Waltham, MS) and the matching secondaries for eYFP (goat anti chicken, 1:200, 103545155, Dianova, Hamburg) , PV (same as above), CR (donkey anti goat, 1:200, A11058, Invitrogen) and CB (donkey anti rabbit, 1:200, A1042, Invitrogen).
Validation	Nuclear counterstains were done with Hoechst (H1399, Thermo Fisher Scientific).

Animals and other research organisms

Policy information about studies involving animals; ARRIVE guidelines recommended for reporting animal research, and Sex and Gender in Research

Laboratory animals	Zebra finch (Taeniopygia guttata), older than 120 dph, randomly selected from the breeding colony at the Max Planck Institute for Biological Intelligence in Seewiesen
Wild animals	The study did not involve wild animals.
Reporting on sex	The findings only apply to male zebra finches because female zebra finches do not learn to sing due to a dimorphims in their brain architecture.
Field-collected samples	This study did not involve samples collected in the field.
Ethics oversight	The Government of Upper Bavaria: licenses AZ 02-19-153 and AZ 02-19-185

Note that full information on the approval of the study protocol must also be provided in the manuscript.

Plants

Seed stocks	*Report on the source of all seed stocks or other plant material used. If applicable, state the seed stock centre and catalogue number. If plant specimens were collected from the field, describe the collection location, date and sampling procedures.*
Novel plant genotypes	*Describe the methods by which all novel plant genotypes were produced. This includes those generated by transgenic approaches, gene editing, chemical/radiation-based mutagenesis and hybridization. For transgenic lines, describe the transformation method, the number of independent lines analyzed and the generation upon which experiments were performed. For gene-edited lines, describe the editor used, the endogenous sequence targeted for editing, the targeting guide RNA sequence (if applicable) and how the editor was applied.*
Authentication	*Describe any authentication procedures for each seed stock used or novel genotype generated. Describe any experiments used to assess the effect of a mutation and, where applicable, how potential secondary effects (e.g. second site T-DNA insertions, mosiacism, off-target gene editing) were examined.*

This checklist template is licensed under a Creative Commons Attribution 4.0 International License, which permits use, sharing, adaptation, distribution and reproduction in any medium or format, as long as you give appropriate credit to the original author(s) and the source, provide a link to the Creative Commons license, and indicate if changes were made. The images or other third party material in this article are included in the article's Creative Commons license, unless indicated otherwise in a credit line to the material. If material is not included in the article's Creative Commons license and your intended use is not permitted by statutory regulation or exceeds the permitted use, you will need to obtain permission directly from the copyright holder. To view a copy of this license, visit http://creativecommons.org/licenses/by/4.0/
